# Decoding pan-cancer treatment outcomes using multimodal real-world data and explainable artificial intelligence

**DOI:** 10.1038/s43018-024-00891-1

**Published:** 2025-01-30

**Authors:** Julius Keyl, Philipp Keyl, Grégoire Montavon, René Hosch, Alexander Brehmer, Liliana Mochmann, Philipp Jurmeister, Gabriel Dernbach, Moon Kim, Sven Koitka, Sebastian Bauer, Nikolaos Bechrakis, Michael Forsting, Dagmar Führer-Sakel, Martin Glas, Viktor Grünwald, Boris Hadaschik, Johannes Haubold, Ken Herrmann, Stefan Kasper, Rainer Kimmig, Stephan Lang, Tienush Rassaf, Alexander Roesch, Dirk Schadendorf, Jens T. Siveke, Martin Stuschke, Ulrich Sure, Matthias Totzeck, Anja Welt, Marcel Wiesweg, Hideo A. Baba, Felix Nensa, Jan Egger, Klaus-Robert Müller, Martin Schuler, Frederick Klauschen, Jens Kleesiek

**Affiliations:** 1 Institute for Artificial Intelligence in Medicine, University Hospital Essen (AöR), Essen, Germany; 2 Institute of Pathology, University Hospital Essen (AöR), Essen, Germany; 3 Institute of Pathology, Ludwig-Maximilians-University Munich, Munich, Germany; 4 BIFOLD – Berlin Institute for the Foundations of Learning and Data, Berlin, Germany; 5 Machine Learning Group, Technical University of Berlin, Berlin, Germany; 6 Department of Mathematics and Computer Science, Freie Universität Berlin, Berlin, Germany; 7 Institute for Diagnostic and Interventional Radiology and Neuroradiology, University Hospital Essen (AöR), Essen, Germany; 8 Department of Medical Oncology, University Hospital Essen (AöR), Essen, Germany; 9 Medical Faculty, University of Duisburg-Essen, Essen, Germany; 10 West German Cancer Center, University Hospital Essen (AöR), Essen, Germany; 11 German Cancer Consortium (DKTK), Partner site University Hospital Essen (AöR), Essen, Germany; 12 Department of Ophthalmology, University Hospital Essen (AöR), Essen, Germany; 13 Department of Endocrinology, Diabetes and Metabolism, University Hospital Essen (AöR), Essen, Germany; 14 Division of Clinical Neurooncology, Department of Neurology and Center for Translational Neuro- and Behavioral Sciences (C-TNBS), University Medicine Essen, University Duisburg-Essen, Essen, Germany; 15 Department of Urology, University Hospital Essen (AöR), Essen, Germany; 16 Department of Nuclear Medicine, University Hospital Essen (AöR), Essen, Germany; 17 Department of Gynecology and Obstetrics, University Hospital Essen (AöR), Essen, Germany; 18 Department of Otorhinolaryngology, University Hospital Essen (AöR), Essen, Germany; 19 Department of Cardiology and Vascular Medicine, West German Heart and Vascular Center Essen, University Hospital Essen (AöR), Essen, Germany; 20 Department of Dermatology, University Hospital Essen (AöR), Essen, Germany; 21 Research Alliance Ruhr, Research Center One Health, University of Duisburg-Essen, Essen, Germany; 22 Bridge Institute of Experimental Tumor Therapy, West German Cancer Center, University Hospital Essen (AöR), University of Duisburg-Essen, Essen, Germany; 23 Division of Solid Tumor Translational Oncology, German Cancer Consortium (DKTK Partner Site Essen) and German Cancer Research Center, DKFZ, Heidelberg, Germany; 24 Department of Radiotherapy, University Hospital Essen (AöR), Essen, Germany; 25 Department of Neurosurgery and Spine Surgery, University Hospital Essen (AöR), Essen, Germany; 26 Department of Artificial Intelligence, Korea University, Seoul, South Korea; 27 MPI for Informatics, Saarbrücken, Germany; 28 German Cancer Consortium (DKTK), German Cancer Research Center (DKFZ), Berlin partner site, Berlin, Germany; 29 German Cancer Consortium (DKTK), German Cancer Research Center (DKFZ), Munich partner site, Munich, Germany; 30 Bavarian Cancer Research Center (BZKF), Erlangen, Germany

**Keywords:** Tumour biomarkers, Prognostic markers, Translational research, Cancer, Machine learning

## Abstract

Despite advances in precision oncology, clinical decision-making still relies on limited variables and expert knowledge. To address this limitation, we combined multimodal real-world data and explainable artificial intelligence (xAI) to introduce AI-derived (AID) markers for clinical decision support. We used xAI to decode the outcome of 15,726 patients across 38 solid cancer entities based on 350 markers, including clinical records, image-derived body compositions, and mutational tumor profiles. xAI determined the prognostic contribution of each clinical marker at the patient level and identified 114 key markers that accounted for 90% of the neural network’s decision process. Moreover, xAI enabled us to uncover 1,373 prognostic interactions between markers. Our approach was validated in an independent cohort of 3,288 patients with lung cancer from a US nationwide electronic health record-derived database. These results show the potential of xAI to transform the assessment of clinical variables and enable personalized, data-driven cancer care.

## Abstract (web summary)

Keyl et al. present an explainable artificial intelligence model-based real-world data analysis from over 15,000 patients across 38 cancer types, identified key prognostic marker interactions, and confirmed these in an external lung cancer cohort.

## Main

Despite the vast amount of multimodal clinical data currently available for each patient in modern healthcare, the promise of personalized medicine has yet to be realized. Single-marker studies do not provide sufficient insight into the complex interplay of patient- and tumor-specific variables that determine a patient’s prognosis^1^. As a result, many of the proposed tools are not used in clinical practice or do not consider the patient’s entire clinical data reflecting the unique disease context^2,3^. A promising strategy to overcome this limitation is to integrate clinical data from multiple sources, such as medical history, laboratory test results, imaging data and omics analyses^1,4^. Advances in machine learning and the increasing availability of digitally accessible data made it possible to model complex relationships between prognostic markers on a large scale^1,5–9^. Together with recent methods for understanding the decision-making of such models, referred to as explainable artificial intelligence (xAI), this makes it possible to assess individual patient prognosis and unravel the contribution of each variable^10–15^.

In this study, we leveraged these advances by proposing an approach for decoding prognostic hallmarks based on large-scale real-world data (RWD). We modeled patient outcomes using a deep neural network and applied the xAI method layer-wise relevance propagation (LRP) to disentangle how each piece of clinical information contributed to an individual patient’s prognosis^5,12^. Our dataset comprises multimodal data from 15,726 patients across 38 cancer entities undergoing systemic treatment. The data include clinical examination, laboratory tests, clinical records, computed tomography (CT) imaging-derived body composition and genetic data.

Until now, many existing clinical predictors have been cancer-entity specific and not designed to incorporate cross-cancer associations. However, available data suggest that similarities between patients extend beyond the histological tumor type, leading to an increasing number of trials that include patients with different cancer entities^16–21^.

Training our deep-learning approach on a pan-cancer dataset enabled the neural network to learn prognostic relationships that extend across cancer entities. This facilitates the development of a comprehensive model that reveals clinically relevant biomarker signatures without prior knowledge. As a result, our approach can aid clinicians in prioritizing critical patient-specific information and optimizing therapeutic strategies. This approach paves the way for transparent xAI-guided decision-making compliant with legal requirements^22^. We confirmed the reproducibility and validity of this xAI approach on an external real-world dataset comprising 3,288 patients with lung cancer from a US nationwide, electronic health record-derived deidentified database.

The growing abundance and accessibility of RWD is increasingly revealing its potential for clinical application. In this study, we move further and demonstrate the ability of xAI to decode patient outcomes and provide tailored treatment guidance based on multimodal RWD.

## Results

### Cohort definition

**Fig. 1:**
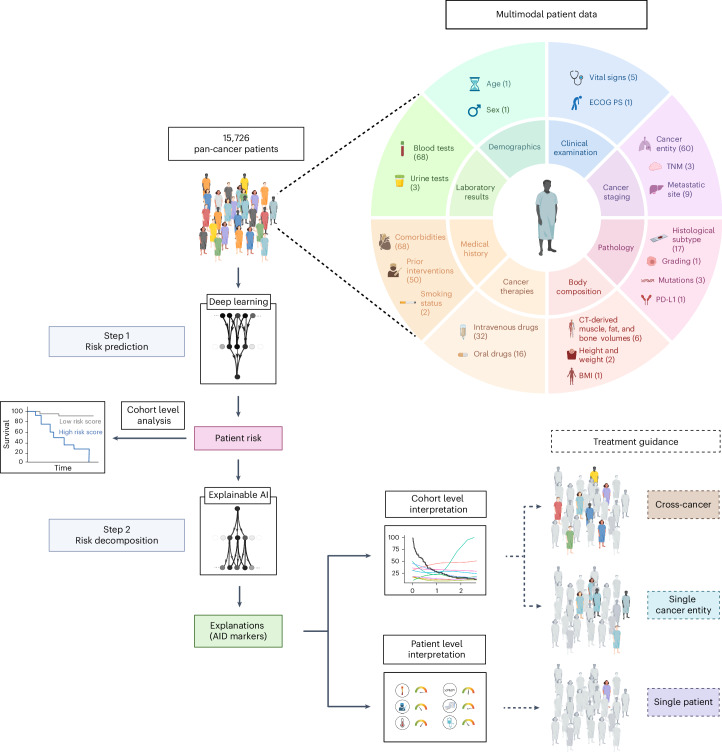
Overview of the data composition and explainable AI (xAI)-based workflow for decoding treatment outcomes. Following the collection of multimodal pan-cancer data, each patient’s risk score is predicted by deep learning and enables patient stratification. xAI then decomposes the patient risk into the individual contributions of each marker. This enables treatment guidance at the patient and cohort level. The numbers in parentheses indicate the number of variables for each data type.

**Extended Data Fig. 1:**
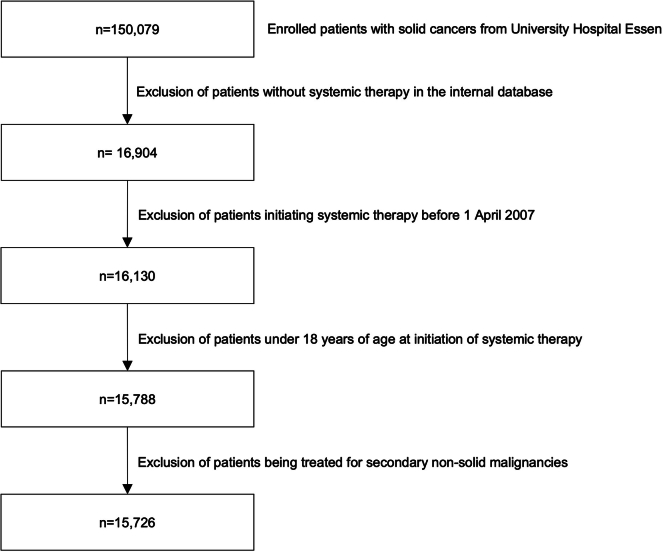
Patient inclusion. Flowchart showing the process of patient inclusion.

We retrospectively evaluated data from 150,079 patients with cancer with available medical records treated at the West German Cancer Center of the University Hospital Essen, one of Germany’s largest academic comprehensive cancer centers. Of these, 15,726 patients (44.3% female) who received systemic cancer treatment between April 2007 and July 2022 (median: November 2016) were included in the final analysis (Extended Data Fig. 1). The most frequent cancer entities were lung cancer (*n* = 4,320), sarcoma (*n* = 1,578) and breast cancer (*n* = 1,223; for details, see Supplemental Table 1). Censoring was performed on 7,349 patients (46.7%) to calculate overall survival (OS) and on 5,638 patients (35.9%) to calculate time to next treatment (TTNT). Metastatic status (M status) was available in a structured format at baseline for 7,965 patients. Of those, 5,606 patients were treated for metastatic disease (M1), and 2,359 patients received systemic therapy for localized or locally advanced cancers (M0). In 5,395 patients, body composition was automatically assessed from abdominal CT images taken before treatment initiation^23,24^. In total, we included 350 variables in our analysis, consisting of different modalities and both patient- and tumor-specific variables, providing a detailed patient characterization before the first systemic treatment at our institute (Fig. 1).

### Development of pan-cancer models for outcome prediction

**Extended Data Fig. 2:**
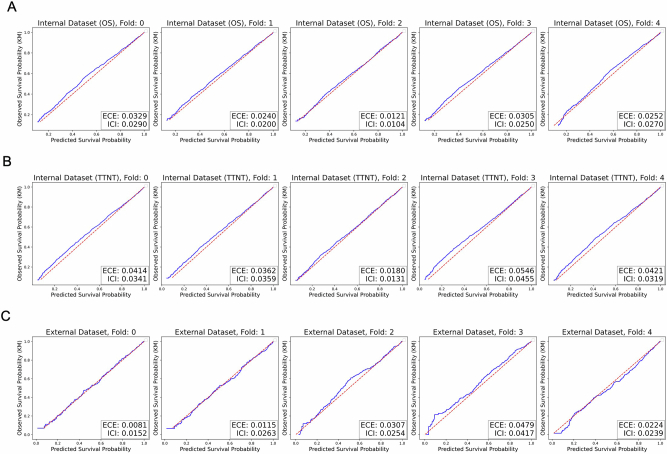
Calibration results. Calibration plots showing the relationship between average predicted survival probability (x axis) and observed survival probability (via Kaplan-Meier fitter) on the test set. a: Internal dataset (OS), b: Internal dataset (TTNT), c: External dataset (OS). ECE: Expected calibration error, ICI: Integrated calibration index. Source data

Two neural networks were trained to predict OS or TTNT for each patient based on their medical profile at the time of first in-house systemic treatment. We demonstrated the reliability of the neural networks by performing a five-fold cross-validation for OS and TTNT prediction, respectively. For each fold, 80% of the data were used for training the neural network, 10% for hyperparameter tuning and 10% for testing. Calibration results are shown in Extended Data Fig. 2.

**Fig. 2:**
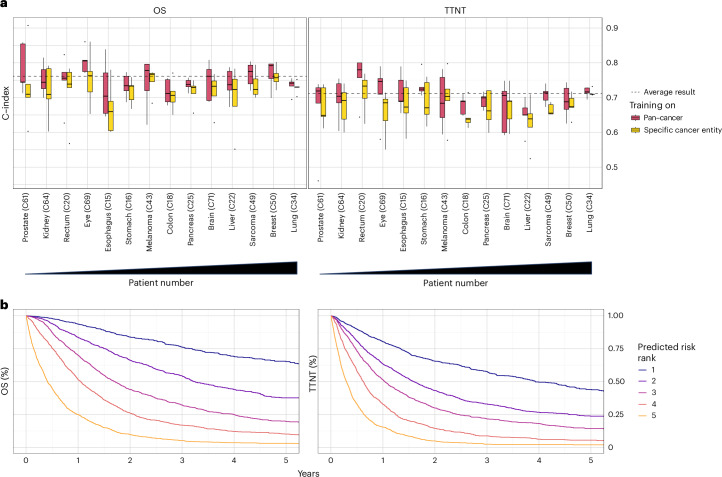
Prediction of prognosis following training on pan-cancer RWD. **a**, Concordance index for predicting OS and TTNT in five-fold cross-validation. The dashed line indicates the prediction result over all patients averaged across folds. Box plots show prediction results for individual cancer entities with at least 20 patients in the test set (*n* = 6,070 patients overall; prostate: *n* = 131; kidney: *n* = 147; eye: *n* = 187; esophagus: *n* = 198; rectum: *n* = 199; stomach: *n* = 300; pancreas: *n* = 304; brain: *n* = 312; colon: *n* = 319; melanoma: *n* = 324; liver: *n* = 373; sarcoma: *n* = 538; breast: *n* = 619; lung: *n* = 2,119) of each fold after training the neural network on all cancer entities (red) or the specific cancer entity (yellow). Cancer entities are ordered from left to right by ascending patient numbers in the overall dataset. Median is indicated by center line, bounds of boxes indicate interquartile range, and whiskers extend to a maximum distance of 1.5 ⋅ IQR from the hinge. Data beyond the end of whiskers are plotted individually. **b**, Kaplan-Meier plots for OS and TTNT in the pan-cancer dataset for patients of the combined test sets (*n* = 7,861) patients. Patients were stratified into five risk groups according to the risk predicted by the (pan-cancer trained) neural network. Source data

The survival model achieved an average concordance index (C-index) on the pan-cancer dataset of 0.762 (range across folds: 0.758–0.764) for OS prediction and 0.711 (range: 0.702–0.718) for TTNT prediction of patients across all cancer entities (Fig. 2a). When the model performance was tested independently for each cancer entity with at least 20 patients in each fold’s test set, the predictive performance varied. For OS, the highest C-index was achieved for ocular cancers (0.804, range: 0.771–0.860), whereas the highest C-index of TTNT was achieved for rectal cancers (0.756, range: 0.644–0.800).

Training models on the pan-cancer dataset, as opposed to exclusively training on single cancer entities, significantly improved model performance for both OS (mean C-index of patients within individual cancer entities: 0.75 versus 0.72, *P* < 0.001) and TTNT (mean C-index of patients within individual cancer entities: 0.70 versus 0.68, *P* < 0.001). Only in melanoma patients, the mean results (mean C-index for OS: 0.74 versus 0.75, mean C-index for TTNT: 0.69 versus 0.7, *P* > 0.05) were better when the training was performed on the melanoma cohort compared to training on the pan-cancer cohort. The advantage of the pan-cancer model over the single-entity models suggests that it used prognostic information shared by the overall cohort to provide robust predictions.

After training on a large and granular real-world pan-cancer dataset, both neural networks for predicting OS and TTNT were able to stratify patients from the test sets into distinct cross-cancer risk groups (Fig. 2b).

**Fig. 3:**
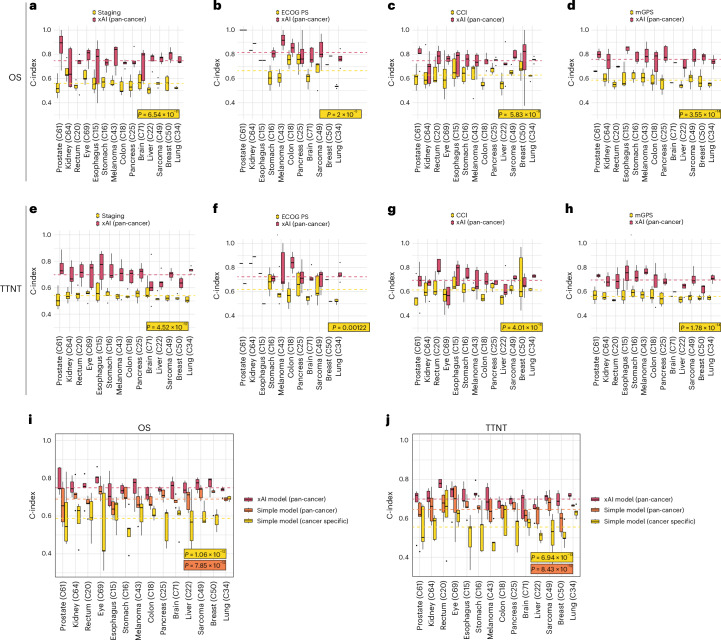
Benchmarking xAI against common clinical prognostic approaches. **a**–**h**, Filtered for patients for whom clinical markers were present. Lines indicate the average of all C-indices calculated for each fold and cancer type. **a**,**e**, UICC Staging (*n* = 7,572 patients, *P* = 6.54 × 10^−11^ and 4.52 × 10^−12^). **b**,**f**, Eastern Cooperative Oncology Group performance status (ECOG PS) (*n* = 2,035 patients, *P* = 2 × 10^−5^ and 0.00122). **c**,**g**, Charlson Comorbidity Index (CCI; *n* = 7,965 patients, *P* = 5.83 × 10^−9^ and 4.01 × 10^−6^). **d**,**h**, Modified Glasgow prognostic score (mGPS; *n* = 6,042 patients, *P* = 3.55 × 10^−14^ and 1.78 × 10^−14^). **i**,**j**, Comparison between the pan-cancer xAI model and a parsimonious Cox model trained on all patients or on patients with the test set tumor type for OS (**i**, *n* = 6,070 patients, *P* = 1.06 × 10^−12^ and 7.85 × 10^−12^) and TTNT (**j**, *n* = 6,070 patients, *P* = 6.94 × 10^−13^ and 8.43 × 10^−12^). Median is indicated by center line, bounds of boxes indicate interquartile range and whiskers extend to a maximum distance of 1.5 ⋅ IQR from the hinge. Data beyond the end of whiskers are plotted individually. *P* values are derived from Wilcoxon ranked test (two sided). Source data

We compared the performance of the pan-cancer models against common prognostic scores (Fig. 3a–h). Reporting the average C-index, the xAI model outperformed UICC Staging (OS: 0.75 versus 0.56, *P* < 0.001; TTNT: 0.70 versus 0.54, *P* < 0.001), the Eastern Cooperative Oncology Group Performance Status (ECOG PS; OS: 0.81 versus 0.67, *P* < 0.001, TTNT: 0.72 versus 0.62, *P* = 0.001), the Charlson Comorbidity Index (CCI, OS: 0.75 versus 0.63, *P* < 0.001, TTNT: 0.69 versus 0.61, *P* < 0.001) and the modified Glasgow prognostic score (mGPS, OS: 0.76 versus 0.59, *P* < 0.001, TTNT: 0.70 versus 0.56, *P* < 0.001).

For clinical deployment, a small set of variables would facilitate the application of models. Therefore, we compared the xAI model to a simplified Cox model fitted on ten automatically selected variables (Fig. 3i,j). The pan-cancer xAI model outperformed the simplified model when fitted on the complete training dataset (average C-index: 0.75 versus 0.69, *P* < 0.001) and when fitted on the respective cancer type (average C-index: 0.75 versus 0.59, *P* < 0.001).

### xAI reveals complex prognostic relationships between markers

After developing reliable outcome prediction models, we applied xAI to unravel how clinical information of individual patients influences the neural networks in assessing prognosis. We chose to explain the pan-cancer models since they outperformed cancer-specific models overall. We selected the xAI method layer-wise relevance propagation (LRP) because it allows for the computation of robust explanations at low computational cost for individual patients^12^. LRP computed for each patient the risk contribution (RC) of every clinical variable, such as laboratory markers or comorbidities, to the predicted favorable or unfavorable outcome. This results in AI-derived (AID) markers with two dimensions, the original marker value and its LRP-assigned RC. A positive RC indicates a contribution to an adverse outcome and a negative RC indicates a contribution to a favorable outcome.

**Fig. 4:**
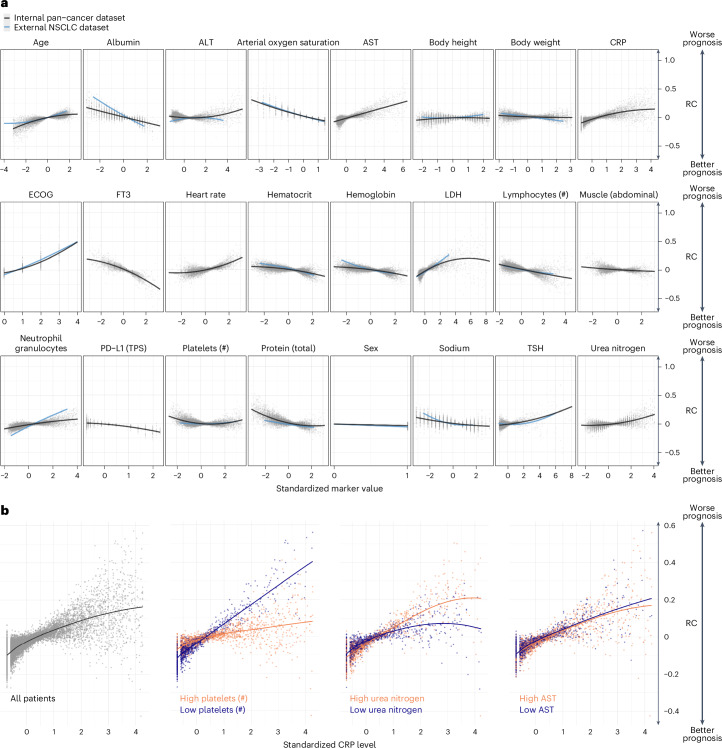
Contribution of clinical markers to the prediction of OS. **a**, Marker RC on the OS prediction. Each point represents one marker value for one patient versus the LRP-assigned RC (*y* axis) to the patient’s prognosis. Marker values are standardized. **b**, RC of CRP depended on the value of other markers. The left plot shows the standardized CRP level and LRP-assigned RC for all patients. The right three plots depict the patients for whom the three selected markers: platelet count, urea nitrogen and AST, were in the highest or lowest 10% quantile. Source data

By analyzing the AID markers across all patients, it was possible to investigate how the neural network evaluated the relationship between the marker and its contribution to the patient’s risk (Fig. 4a). For example, increasing age and elevated levels of C-reactive protein (CRP) strongly contributed to predicting an unfavorable prognosis. In contrast, high fT3, high PD-L1 TPS and higher CT-derived abdominal muscle volume contributed to predicting a favorable prognosis.

**Extended Data Fig. 3:**
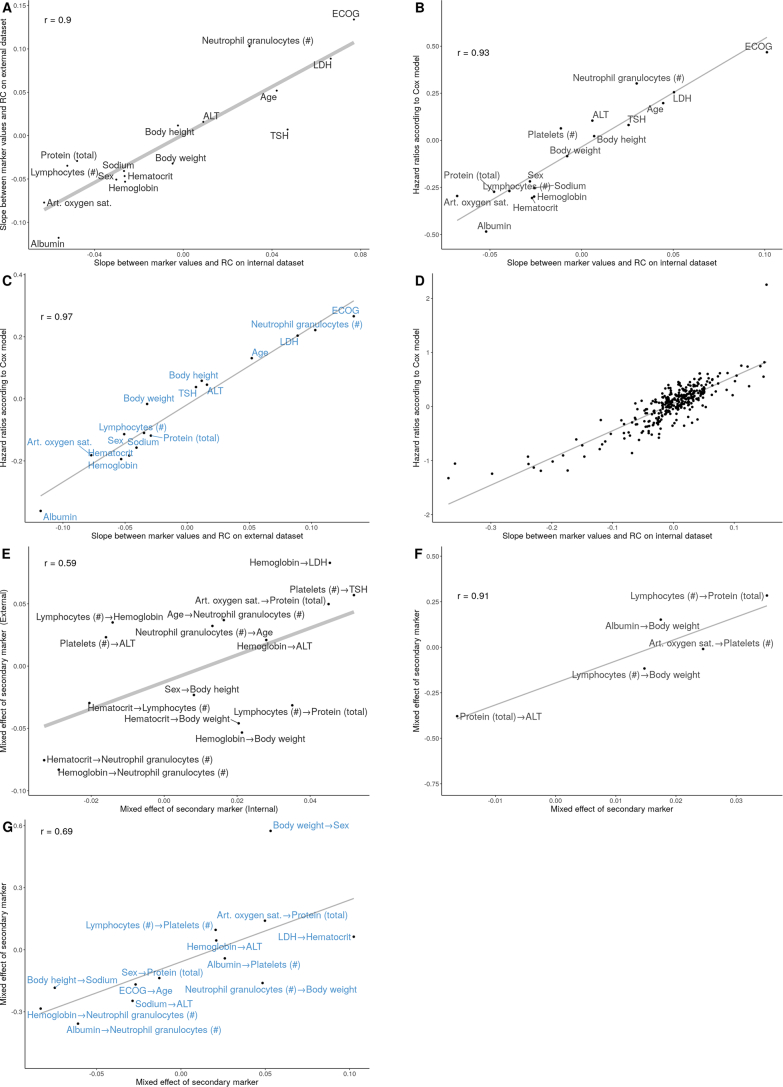
Replicability of the xAI approach and comparison to linear methods. **A:** Replicability of the xAI approach on the external dataset. Axes indicate the (linearized) relationship between marker values and their xAI-assigned RCs for the Internal (x axis) and external (y axis) datatset. **B,C:** Validation of xAI results with Cox regression models. The x axis shows the linearized relationships between marker values and RC according to xAI. The y axis shows the hazards of each marker according to a univariate cox regression model on the same dataset (B: Internal data, C: External data). **D:** Validation of xAI results with Cox regression models (all markers, Pearson’s r = 0.85). **E:** Comparison of higher order interactions identified by xAI between internal (x axis) and external (y axis) dataset. Given the linearized relationship between a marker Y and the RC of Y, the label X- > Y defines how this relationship changes between patient groups with high and low X. **F, G:** Complex interactions found by xAI can be validated with mixed-effects Cox proportional hazards models. The effects captured by xAI (x axis) correspond strongly to the effects estimated by mixed-effects Cox proportional hazards models (F: Internal data, G: External data). Source data

We validated the results for a subset of markers using external data from 3,288 patients with non-small cell lung cancer (NSCLC) provided by Flatiron Health. Upon applying our approach to the external dataset, we found a strong correlation between the linearized slopes of RCs on the internal and external datasets (Pearson’s *r* = 0.9, *P* < 0.001; Extended Data Fig. 3a). Thus, xAI predicted a comparable impact of markers on patient risk in both datasets. To confirm if the fundamental results of LRP matched conventional models, we examined the simplified linearized effect predicted by xAI against a standard Cox proportional hazards model. Our analysis revealed that the relationships computed on the internal and external datasets strongly correlated to the hazard ratios of each marker (subset of markers measured in both datasets: internal dataset: Pearson’s *r* = 0.93, *P* < 0.001, external dataset: Pearson’s *r* = 0.97, *P* < 0.001, Extended Data Fig. 3b,c; all markers in internal dataset: Pearson’s *r* = 0.85, *P* < 0.001, Extended Data Fig. 3d).

Notably, the RC of a marker varied widely even when different patients had the same marker value. By utilizing LRP, it becomes possible to explain some of the variance in RC by marker interactions (Fig. 4b). We observed how the RC of CRP varied depending on the values of additional ‘secondary’ variables. Out of 8,294 examined marker pairs, 1,373 (16.6%) showed significant interactions according to a mixed-effects model. For example, high CRP levels were assigned a high RC, particularly when platelet counts were low (Δ RC slopes: ×0.07, *P* < 0.001). CRP had less influence on the predicted risk when the platelet count was high. Although the prognostic significance of elevated CRP levels and platelet counts is known, the exact interaction has not yet been described^25^. The impact of blood urea nitrogen (BUN) on the RC of CRP was less pronounced (Δ RC slopes: 0.03, *P* < 0.001). Here, a higher CRP level was associated with a particularly high RC in patients with high BUN levels. In contrast, the RC of CRP was independent of aspartate aminotransferase (AST) (Δ RC slopes: −0.006, *P* = 1.0).

The statistically significant interactions between the variables present in the internal and external datasets showed a high level of similarity in the external dataset (Pearson’s *r* = 0.59, *P* = 0.021; Extended Data Fig. 3e). To confirm that the fundamental interaction results observed with xAI were consistent with conventional models, we examined the simplified linearized effect over the LRP-assigned RC against a mixed-effects Cox proportional hazards model.

Here, the direction of interactions derived from xAI matched the interactions observed with the Cox regression models in the internal and external datasets (*r* = 0.91, *P* = 0.03 and *r* = 0.69, *P* = 0.009; Extended Data Fig. 3f,g). Based on these results, we concluded that the LRP approach was highly reproducible across various datasets as well as consistent with established statistical models that simplify relationships. However, the xAI approach’s full potential extends beyond this and enables nonlinear RC assignments for individual patients, taking into account their unique disease context.

**Extended Data Fig. 4:**
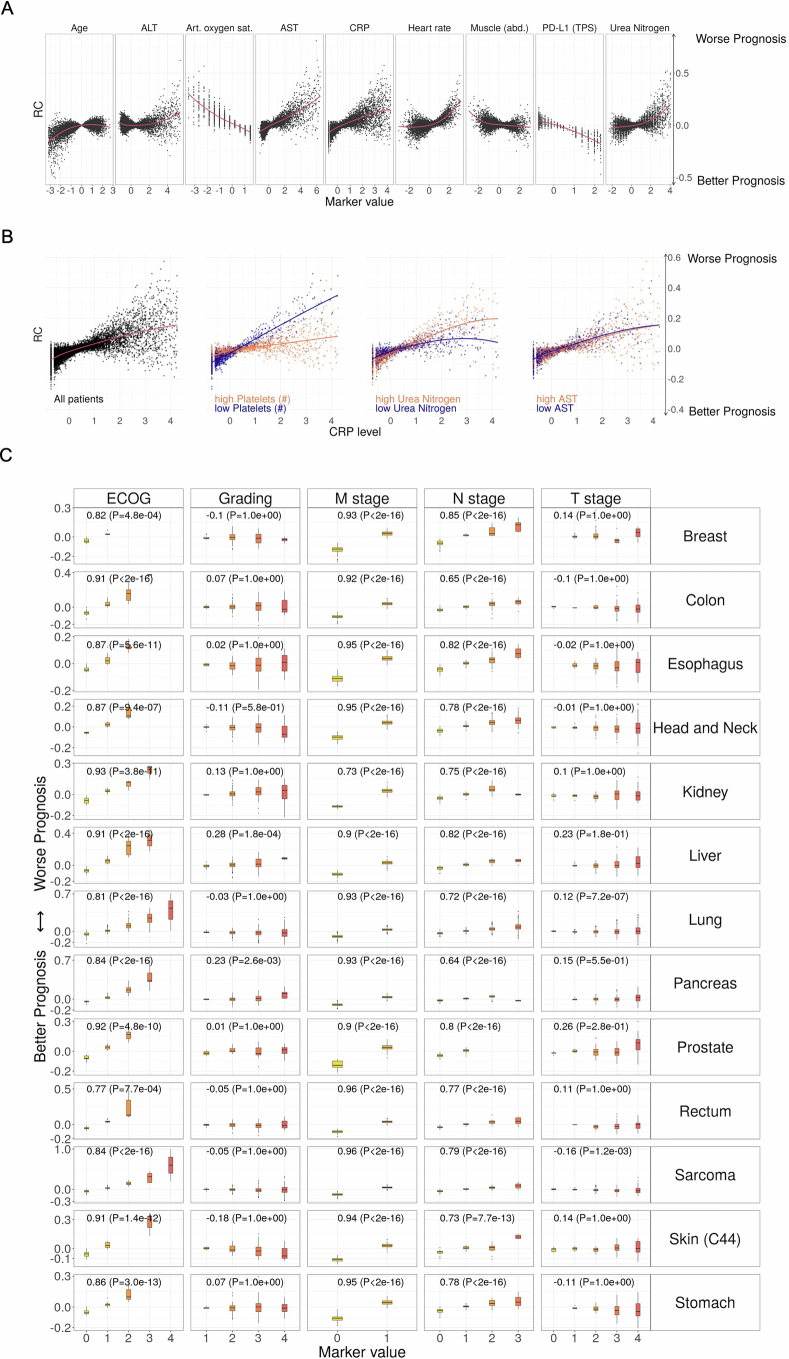
Prognostic value of selected markers. **A:** Marker risk contribution (RC) on the TTNT prediction. Each point represents one marker value for one patient versus the LRP-assigned RC (y-axis) to the patient’s prognosis. Marker values are standardized. **B:** The risk contribution of CRP depended on the value of other markers. The standardized CRP level and LRP-assigned RC are shown for all patients in the left plot. The right three plots depict the patients for whom the three selected markers platelet count, urea nitrogen and AST were in the highest or lowest 10% quantile. **C:** Comparison of established prognostic scores with the LRP-assigned RC for OS (n = 7,196 patients). The x-axis depicts the value of the different scores. The y-axis indicates the RC. Comparison is shown for each marker and cancer type. Cancer entities are shown only if the respective marker has been measured in at least 20 patients. Adjusted P values are shown in brackets (two-sided, Pearson’s correlation, Holms correction). Adjusted P values for ECOG PS were 4.78e-04, 6.60e-19, 5.56e-11, 9.42e-07, 3.80e-11, 1.25e-18, 5.89e-242, 5.90e-21, 4.84e-10, 7.75e-04, 2.86e-31, 1.37e-12, and 2.97e-13. For Grading, adjusted P values were 1, 1, 1, 0.58, 1, 0.000178, 1, 0.00256, 1, 1, 1, 1, 1. For M stage, all P values were <2e-16. For N stage, all P values were <2e-16 except for Skin (P = 7.7e-13). For T stage, P values were 1, 1, 1, 1, 1, 0.177, 7.18e-07, 0.549, 0.279, 1, 0.00123, 1, 1. Source data

For results on TTNT, see Extended Data Figure 4a,b.

### AID markers for patient-level treatment guidance

AID markers, the combination of a marker value with its LRP-assigned RC, enhance the clinical information available to healthcare professionals by incorporating the contextual risk associated with each marker. A ‘clinician’s guide’ can clearly present the AID marker profile of individual patients.

**Fig. 5:**
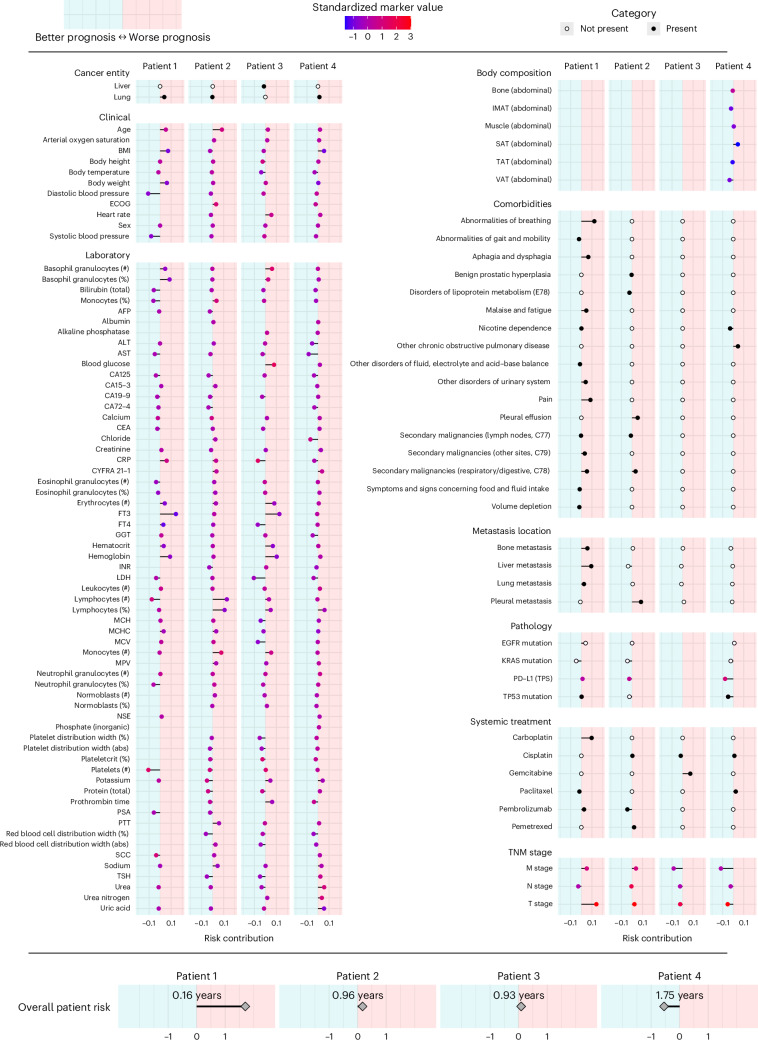
Clinician’s guide showing the contribution of each marker to overall risk at the patient level. Representative results of four patients are presented. The *x* axis indicates the marker’s RC toward higher (right/positive) or lower (left/negative) risk. Colors indicate the presence (black) or absence (white) of cancer entities, comorbidities, metastasis locations and systemic treatment. For markers with ordinal or continuous scales, the point color indicates the marker value for the respective patient. For continuous markers, marker values are standardized. The predicted overall patient risk is displayed at the bottom. To facilitate interpretation, the median absolute survival of 100 patients with a similar predicted risk is given. Body composition markers: abdominal volumes of visceral adipose tissue (VAT), total adipose tissue (TAT), subcutaneous adipose tissue (SAT), intermuscular adipose tissue (IMAT), muscle, bone. Source data

In Fig. 5, we show representative results that illustrate a potential real-world use case of the ‘clinician’s guide’ for four different patients. In patient 1, age, BMI, body weight, and fT3 values contributed unfavorably to the overall prognosis, while the high lymphocyte and platelet counts were assigned a favorable (negative) RC. The patient’s prognosis deteriorated with impaired breathing, aphagia, pain and an advanced T and M stage. Among the different distant metastases, liver metastases were identified as particularly unfavorable compared to lung and bone metastases. Overall, the neural network therefore predicted a highly adverse outcome for this patient based on all available data. In patient 2, lymphocytopenia and older age particularly contributed to a poor prognosis. However, this patient had few comorbidities, with pleural effusion having the strongest unfavorable impact. The absence of liver metastases and the treatment with pembrolizumab were assigned a favorable RC, and the overall risk was considered intermediate. Notably, patient 3 had elevated CRP levels, which is conventionally associated with a potentially dangerous patient condition requiring increased monitoring. However, xAI does not consider this variable to be detrimental in this particular case, possibly because of this patient’s high platelet count and low urea nitrogen levels (Fig. 4). Patient 4 showed medium visceral adipose tissue (VAT), contributing favorably, and low subcutaneous adipose tissue (SAT), contributing adversely. With few comorbidities and no metastases, the overall prognosis was favorable.

### Evaluation of established scoring systems

Our results illustrated the limitations of single marker-based outcome prediction and emphasized the importance of prognostic variables to be considered in the disease context characterized by other markers. In clinical routine, however, it is common to rely on a few scoring systems, such as the TNM stage, to assess prognosis and guide treatment. Based on these scoring systems, patients are usually rigidly categorized, regardless of fundamental differences such as sex, nutritional status or comorbidities.

To evaluate the dependency of a score on this disease context, we analyzed the correlation between the score and the LRP-assigned RC (Extended Data Fig. 4c). For Eastern Cooperative Oncology Group performance status (ECOG PS) (*r* = 0.87), M stage (*r* = 0.92), and N stage (*r* = 0.76), higher scores correlated with higher computed RC on average, indicating a consistent influence on the prognosis independent of other markers. The weak correlation of tumor grade (*r* = 0.02) and T stage (*r* = 0.07) with their RC suggested that they should be interpreted in the context of additional markers.

### Assessment of marker importance at the cohort level

**Extended Data Fig. 5:**
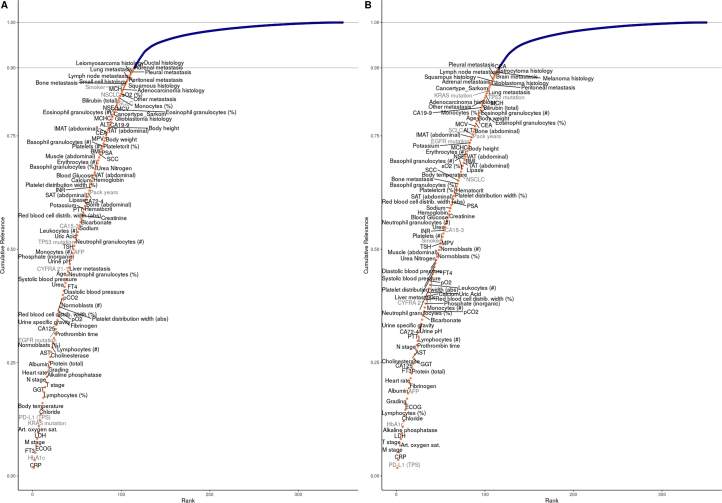
Cumulative relevance for neural network decision-making. **A**: OS, **B**: TTNT. All markers are ranked according to the decreasing marker importance (MI) assigned by LRP across all patients (x axis). MI is corrected for missing values. Y axis shows the cumulative MI. 90 % of all MI is assigned to 114 (TTNT: 115) key prognostic markers. Markers measured in at least 20% of the cancer entities in at least 10% of the patients are shown in black. Source data

**Extended Data Fig. 6:**
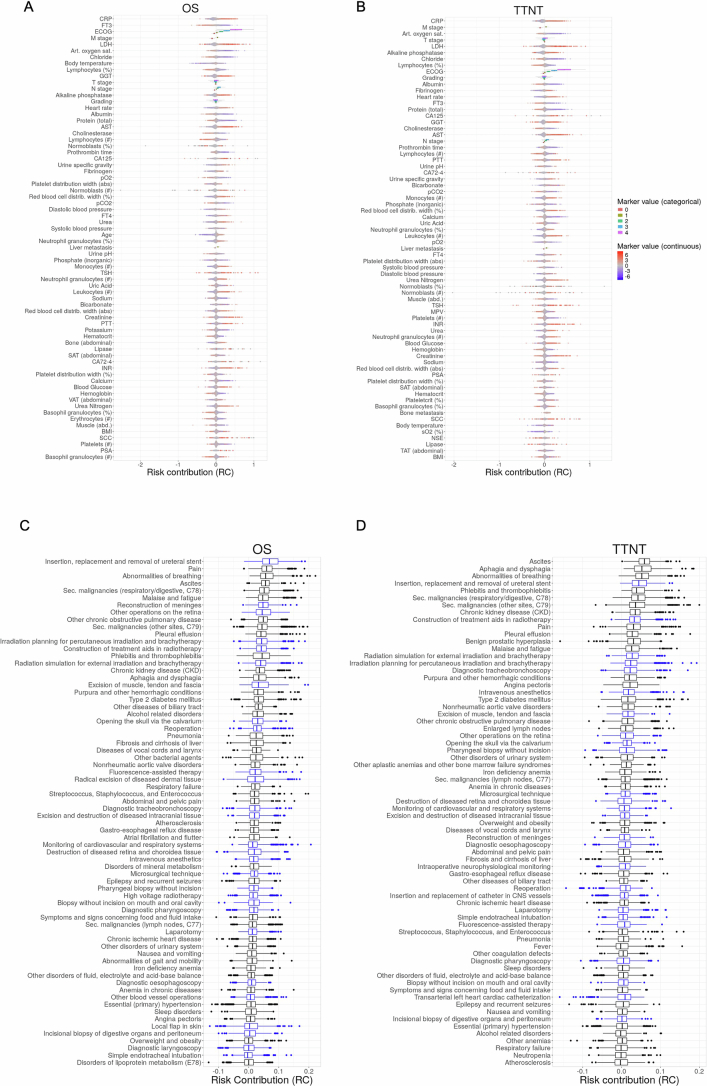
Marker importance. Markers are ordered from top to bottom according to decreasing importance across all patients. **A, B:** Risk contribution (RC) of markers in individual patients is shown on the x axis. RC indicates the contribution to a better (negative) or worse (positive) prognosis. Point color indicates high (red) or low (blue) marker value. (**A**: OS, **B**: TTNT). Cancer entities are shown only if the respective marker has been measured in at least 20 patients. **C, D:** ICD (black) and OPS codes (blue) with the highest assigned RC. **C**: OS (n = 9,713), **D**: TTNT (n = 9,604). Median is indicated by center line, bounds of boxes indicate interquartile range, and whiskers extend to a maximum distance of 1.5 ⋅ IQR from the hinge. Data beyond the end of whiskers are plotted individually. Source data

In a multimodal real-world dataset reflecting clinical care, there are expected to be both sideline markers of low prognostic relevance and critical markers that are highly relevant across patients. To measure the marker importance (MI) in a cohort, we calculated the absolute value of the RC in consistency with other methods in the field^13^. We found that 90% of LRP scores were assigned to the 114 most important markers out of 350 (Extended Data Fig. 5a,b). Across all patients, the most important markers for the prediction of OS were C-reactive protein level (CRP, mean MI: 0.071), free triiodothyronine (fT3, mean MI: 0.066), ECOG PS (mean MI: 0.061), M stage (mean MI: 0.058) and LDH (mean MI: 0.055; Extended Data Fig. 6a,b). These results are consistent with previously reported findings^26–29^. However, our results suggest that fT3 may play a more important role in prognostic assessment than is currently recognized in clinical practice.

Events that are rare in certain cancer subgroups may be common enough in the pan-cancer dataset for models to assess the prognostic impact of the variable. LRP can assess the influence of comorbidities, defined by ICD codes, and medical interventions, defined by the German operation and procedure classification system (OPS), in the disease context (Extended Data Fig. 6c,d). Due to the scarcity of each comorbidity, MI was not informative here, which is why we report the mean RC of affected patients. We found that the comorbidities that contributed the most to the prediction of a poor outcome were pain (mean RC: 0.064), respiratory abnormalities (mean RC: 0.064), ascites (mean RC: 0.056), secondary malignant neoplasm of the respiratory or digestive tract (mean RC: 0.048) and pleural effusion (mean RC: 0.046). Notably, some diagnoses contributed favorably to the overall prognosis (for example, heart failure, gastritis and duodenitis). The interventions that were assigned the highest RC were ureteral stenting (mean RC: 0.074), which may indicate a stenotic process, and meningeal reconstruction (RC: 0.049).

### Cross-cohort comparison of prognostic markers

**Fig. 6:**
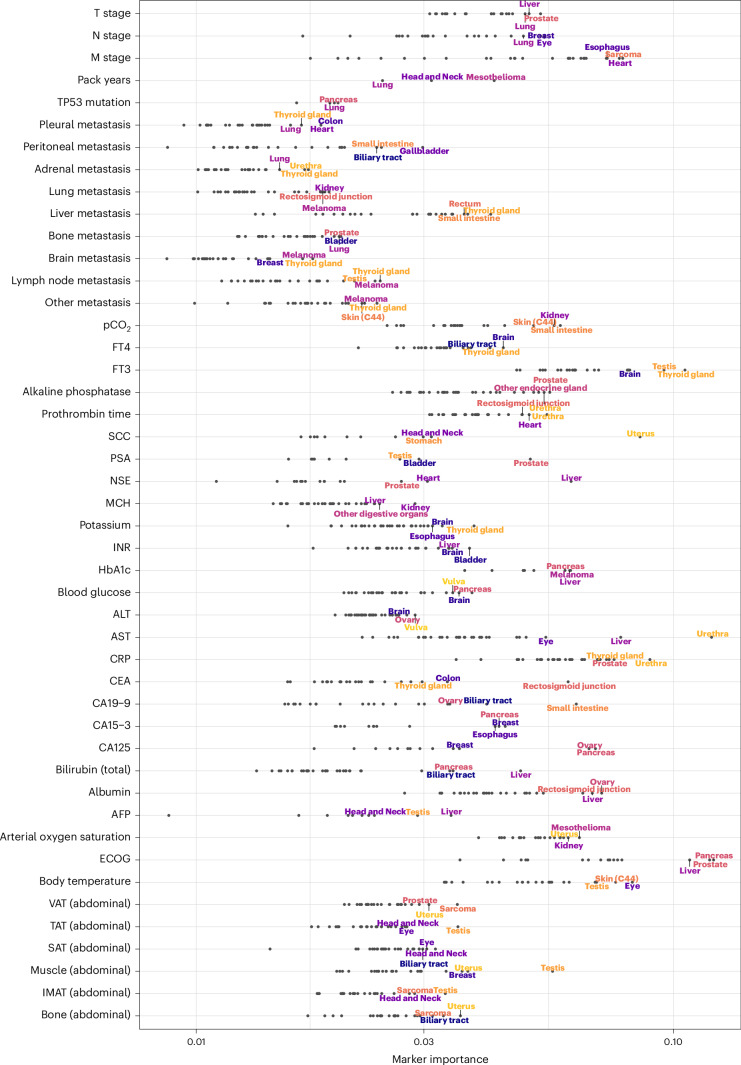
Relationship between mean marker importance (MI) of selected markers and cancer entities. The *x* axis shows the MI on a logarithmic scale. The three cancer entities with the highest marker MI are annotated for each marker. Body composition markers: Abdominal volumes of VAT, TAT, SAT, intermuscular adipose tissue (IMAT), muscle, bone. Cancer entities are shown only if the respective marker has been measured in at least 20 patients. Source data

Model training on a pan-cancer dataset and sample-wise explanations obtained by LRP allowed us to investigate how the MI of a marker differed between patient subgroups (Fig. 6).

Expectedly, LRP identified many markers whose significance in prognosticating a particular cancer type is already established: CA19-9 had the highest MI in cancers of the small intestine, and biliary tract and bilirubin emerged as an essential marker for liver, pancreatic and biliary tract cancers^30–32^. The presence of liver metastases was most relevant for cancers of the thyroid gland, rectosigmoid junction and additional digestive tract cancers^33,34^. HbA1c was most important in cancers of the pancreas and liver^35,36^. The tumor marker CEA had the highest MI in cancers of the rectosigmoid junction, colon and thyroid^37,38^.

However, the cross-cancer approach also made it possible to identify many previously unexplored prognostic associations. Abdominal muscle volume, as determined by CT-based body composition analysis, was most important for vulvar, uterine and testicular cancers. Interestingly, AST had very high MI for urethral cancer, followed by the expected high MI for liver and ocular cancer (mainly uveal melanoma). Alanine transaminase appeared to be most important for the prognostic stratification of patients with cancers of the vulva and ovary. The ECOG PS was particularly important for pancreatic, prostate and liver cancers. Apart from thyroid cancer and brain cancers for which this relationship is well known, fT3 was most important in testicular cancer^39,40^.

**Extended Data Fig. 7:**
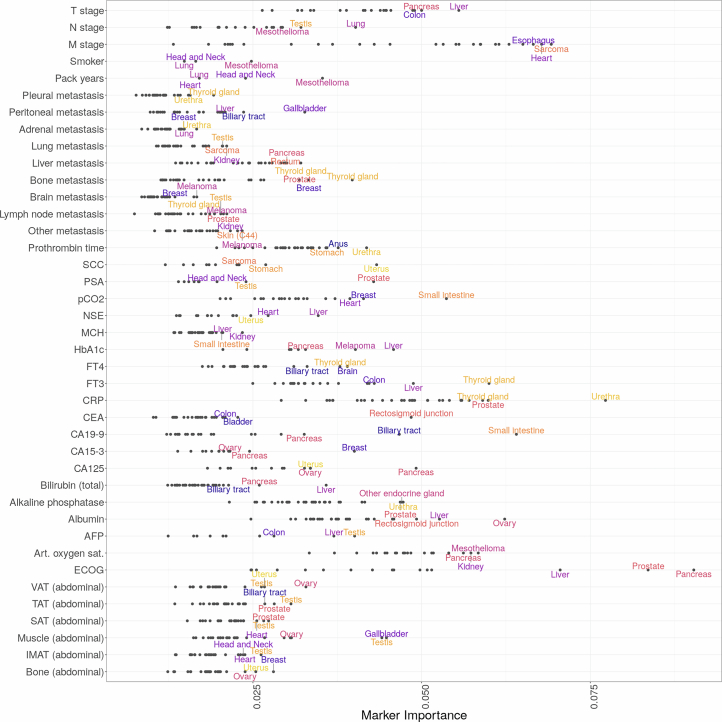
Relationship between marker importance and cancer entities for TTNT. The x axis shows the MI on a logarithmic scale. For each marker, the three cancer entities with the highest marker MI are annotated. Body composition markers: Abdominal volumes of visceral adipose tissue (VAT), total adipose tissue (TAT), subcutaneous adipose tissue (SAT), intermuscular adipose tissue (IMAT), muscle, bone. Source data

For results on TTNT, see Extended Data Fig. 7.

### Evolution of marker importance during disease progression

**Fig. 7:**
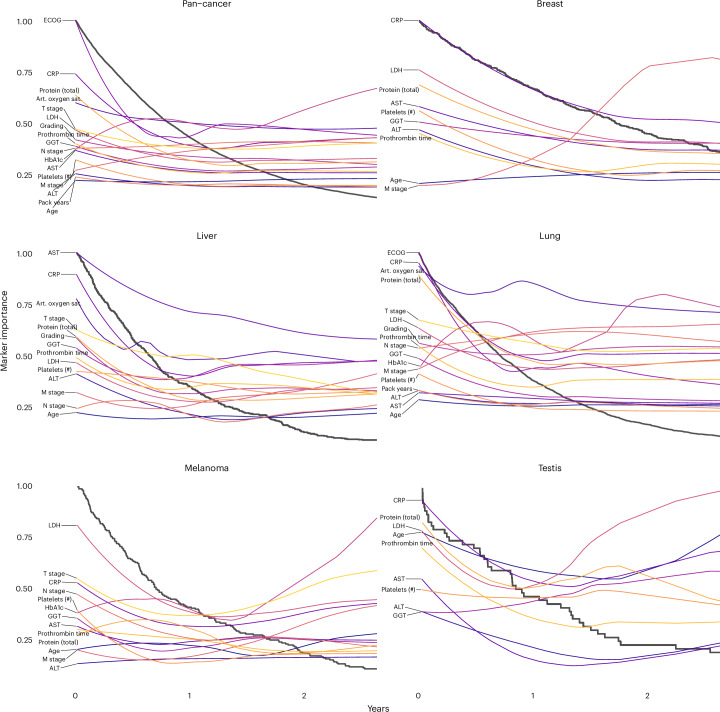
Explainable Kaplan-Meier plots depicting the importance of diagnostic markers during disease progression. Black lines represent Kaplan-Meier plots, whereas the colored lines visualize the change in marker importance (MI) for patients with different survival times. MI lines are scaled between zero and one. Only deceased patients were included in this analysis (pan-cancer: *n* = 8,377, breast: *n* = 487, liver: *n* = 451, lung: *n* = 2,753, melanoma: *n* = 206, testis: *n* = 50). Selected markers were measured in at least 40 patients and within a 2-year window. Art. oxygen sat., arterial oxygen saturation. Source data

Having examined the cancer entity-specific impact of markers on prognosis, we further explored their varying importance for prognostication during disease progression. Ordering the deceased patients according to OS, we could follow the LRP-assigned marker importance along a pseudo timeline and observed distinct changes over the course of treatment (Fig. 7). ECOG PS and CRP and LDH levels were highly prognostic markers throughout disease progression across all cancer entities. The prognosis of patients with a short OS was strongly influenced by total serum protein concentration, which may reflect the relevance of organ dysfunction at this stage of the disease, particularly of the liver and kidneys. The coagulation variable prothrombin time and oxygen saturation were highly prognostic in patients with short OS but contributed much less to the prognosis of patients with long OS. M stage had an overall decisive marker importance, which decreased for disease stages with short OS.

Our modular approach allowed us to generate explainable Kaplan-Meier plots of patient subgroups with different prognoses. In lung cancer, arterial oxygen saturation had the highest MI for most patients, but for patients with short survival, protein expression, CRP and ECOG PS became even more critical. Metastasis (M stage) generally had higher MI than lymph node metastasis and tumor stage. Interestingly, the importance of metastasis decreased during disease progression and was overtaken by T stage and N stage in patients who survived only a few months. LDH had exceptionally high MI in testicular cancer and melanoma, which is well known in the literature^41,42^. The MI of the latter increased during disease progression. In the liver, the MI of AST, total protein, GGT, prothrombin time and LDH increased during disease progression. Alanine transaminase was less important for patients who survived more than one year.

**Extended Data Fig. 8:**
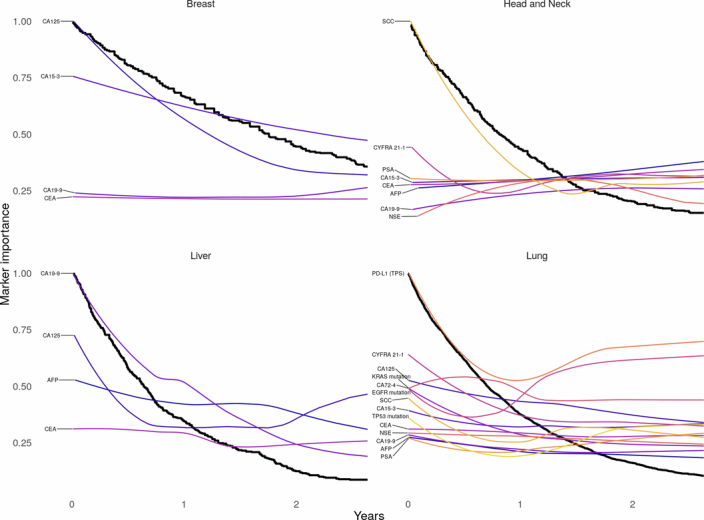
xKM curves for tumor-specific markers (OS). xKM curves show the progress of marker contribution for the prediction of overall survival (OS) for tumor-specific markers along disease progression. Black lines represent Kaplan-Meier plots, while the colored lines visualize the change in marker importance (MI) for patients with different survival times. MI lines are scaled between zero and one. Only deceased patients were included in this analysis (Breast: n = 487, Head and Neck: n = 512, Liver: n = 451, Lung: n = 2,753). Source data

Next, we examined the prognostic impact of cancer-specific biomarkers (Extended Data Fig. 8). PD-L1 TPS was the most important cancer-specific marker for lung cancer prognosis, which aligns with the efficacy of immune checkpoint inhibitor therapy^43^. In head and neck cancer, the tumor marker SCC had a high marker importance that increased during disease progression. In liver cancer, the tumor marker AFP was of high MI throughout disease progression, but CA19-9 and CA125 became more important toward the end of life.

**Extended Data Fig. 9:**
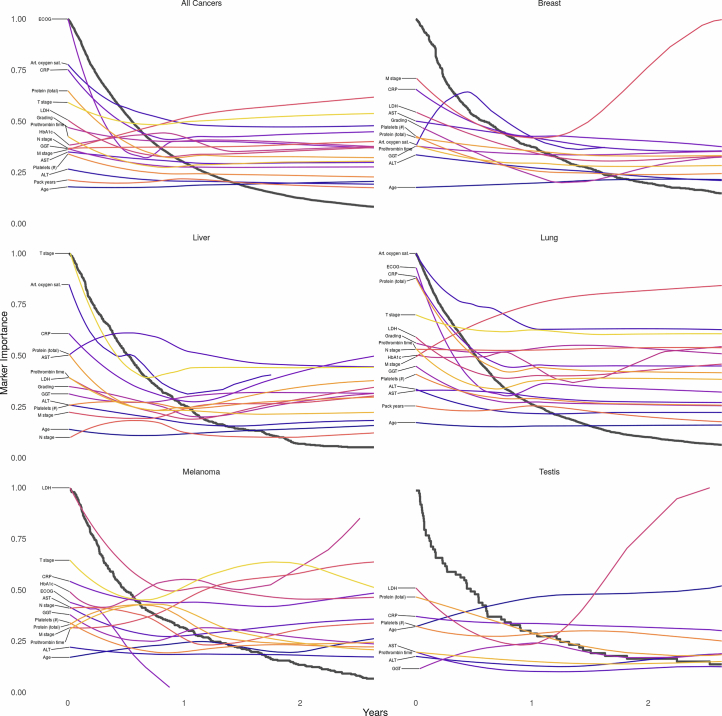
xKM curves for diagnostic markers (TTNT). xKM curves show the progress of marker contribution for the prediction of time-to-next-treatment (TTNT) for markers along disease progression. Black lines represent Kaplan-Meier plots, while the colored lines visualize the change in marker importance (MI) for patients with different survival times. MI lines are scaled between zero and one. Only deceased patients were included in this analysis (Pan-cancer: n = 10,088, Breast: n = 729, Head and Neck: n = 593, Liver: n = 534, Lung: n = 3,105, Testis: 73). Source data

**Extended Data Fig. 10:**
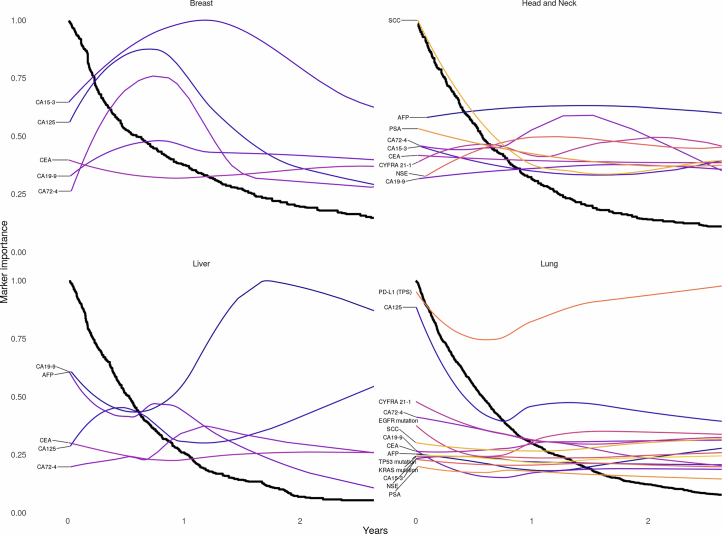
xKM curves for tumor-specific markers (TTNT). xKM curves show the progress of marker contribution for the prediction of time-to-next-treatment (TTNT) for tumor-specific markers along disease progression. Black lines represent Kaplan-Meier plots, while the colored lines visualize the change in marker importance (MI) for patients with different survival times. MI lines are scaled between zero and one. Only deceased patients were included in this analysis (Breast: n = 729, Head and Neck: n = 593, Liver: n = 534, Lung: n = 3,105). Source data

For results on TTNT, see Extended Data Figures 9 and 10.

## Discussion

Personalized medicine requires a comprehensive characterization of individual patients, which cannot be achieved by conventional scoring systems based on limited sets of markers^1,4^. Despite the extensive routine diagnostic data available for each patient, current clinical tools only include small subsets of these variables in a limited number of cancer entities^2,3^. Previous studies have started to show the potential of utilizing multimodal data to predict individual patient prognosis using public databases^7,8,18^. In this study, we utilized multimodal routine clinical data from 15,726 patients with solid cancers undergoing systemic treatment to uncover the complex mechanisms that determine a patient’s prognosis.

Due to the heterogeneity of patients with different cancers and disease stages, we can observe how the influence of specific markers on prognosis changes depending on the individual patient context. We found that the models benefited from training on patients of both the same and different cancer entities, resulting in the successful stratification of patients into cross-cancer risk groups. This is consistent with the growing trend to guide treatment based on predictive biomarkers across cancer entities^19–21^. We assume that these models benefit from the fact that some markers (for example, CRP, ECOG PS) provide similar prognostic information across cancer types, allowing the model to translate learned associations from one cancer entity to another. Using xAI, our study provided a comprehensive understanding of the factors contributing to a treatment outcome. Without using prior knowledge, xAI characterized how each patient’s prognosis was determined by their individual marker profile and identified CRP, fT3, M status and ECOG PS as the most important factors across all patients. Our results showed excellent reproducibility between internal and external datasets and were highly consistent with conventional methods.

In the medical domain, xAI has previously been applied to validate the model performance or assess feature importance across cancer cohorts^18,24,44^. Few studies have made use of patient-wise xAI explanations, which are essential for trusting model decisions and are increasingly required by law for the use of AI systems^18,22^. As the scope of diagnostics increase, it is becoming increasingly difficult for healthcare professionals to integrate all patient information comprehensively. AI-driven treatment guidance has demonstrated its potential to improve patient outcomes^45^. By using xAI and multimodal patient data, our approach goes beyond risk stratification and could simultaneously provide clinicians with AID markers that have dual dimensions, the original marker value and the xAI-assigned RC. This could help healthcare providers and patients adjust treatment intensity and set personalized treatment goals. As patient data can be captured in near-real time within modern hospital infrastructures, our approach could be seamlessly integrated into routine clinical care^46^.

By systematically comparing these AID markers among patients, we show that prognostic associations are not static and that different markers may be critical depending on the cancer entity and the individual disease setting. In contrast to traditional statistical methods, xAI can build on all available data to assess the complex setting of individual patients, provided that common pitfalls are addressed^4,47^.

Confounding is one of the most common challenges in retrospective RWD analysis. We aimed to reduce confounding effects caused by correlated variables by applying high dropout regularization not only to the neural network weights but also to the input to encourage the network to learn variables independently^48^. In a RWD setting, confounding can also be introduced by documentation. For instance, gastritis or duodenitis are not expected to positively impact the patient’s prognosis. However, the documentation of these non-cancer comorbidities may have suggested the absence of an acute life-threatening condition. Also, selection bias should be considered in RWD studies. In this proof-of-concept study, we enrolled only patients receiving systemic cancer therapy. While this cohort provides well-structured treatment data, it is more likely to include patients with advanced disease. The external validation dataset consisted of patients with NSCLC. As NSCLC was the largest cohort in the internal dataset, this was a suitable group for validation, but further external data on different cancer types will need to be included in the future. Particular caution is also needed when interpreting the RC assigned to the different treatments, as the nonrandomized selection of treatments may lead to statistical bias.

In clinical trials, randomization prevents certain forms of confounding and bias. Real-world studies combined with xAI will therefore not replace RCT but may generate new data-driven hypotheses and inform RCT design^49^. Because our approach is not limited to RWD, RCT designed for specific clinical settings could also directly integrate our xAI framework.

In summary, we demonstrate an xAI-based approach for large-scale multimodal data analysis of prognostic relationships in a real-world setting. Given the increasing influence of multimodal data on patient management and therapy selection, xAI approaches hold great potential for precision medicine.

## Methods

### Study design

Electronic health records from 150,079 patients with cancer treated at University Hospital Essen were retrospectively evaluated. Of these, we included 15,726 patients who underwent systemic cancer treatment at University Hospital Essen between 1 April 2007 and 22 July 2022 in this study. OS was defined as the time from initiation of systemic treatment to death from any cause. The date of death was obtained from the medical record or, if unavailable, from the state cancer registry. Patients for whom no date of death was available were censored at the date of the last clinical visit. TTNT was defined as the time from initiation of systemic treatment until initiation of next line of systemic treatment or death from any cause. Patients with no recorded subsequent line of treatment and for whom no date of death was available were censored at the date of the last clinical visit. The study was approved by the Ethics Committee of the Medical Faculty of the University of Duisburg-Essen (No. 21-10347-BO). The requirement for written informed consent was waived due to the retrospective design of the study and the deidentification of data.

### Data acquisition

All medical data were retrieved from the smart hospital information platform (SHIP) of University Hospital Essen. In SHIP, medical data are stored in FHIR format and can be collected based on specific queries. The various subsystems at Essen University Hospital, for example, for laboratory values or electronic medication administration, automatically transfer the data to SHIP. In this study, we created a pan-cancer dataset based on all structured data available in SHIP. First, all patients with solid tumors were collected based on ICD codes (C00-C75). Then, patients who received intravenous or oral cancer treatment documented in SHIP were selected. Further inclusion criteria were initiation of systemic therapy since 1 April 2007 and a minimum age of 18 years at the initiation of cancer treatment. A detailed overview of the patient enrollment process can be found in Extended Data Fig. 1.

For the resulting cohort of 15,726 patients, further clinical data were retrieved from SHIP. To ensure a balance of the most recent data with the fewest missing values in our dataset, we defined different time windows for querying the variable sets relative to the start of systemic cancer treatment. All variables except CT-derived body composition can be mapped to LOINC, SNOMED CT, ATC, ICD or OPS terminologies. Listed below are all of the queried variable sets used to create our dataset, along with the time windows where applicable.

#### Cancer therapies (first recorded in SHIP)

For each patient, the substances of the first line of therapy administered in our cancer center were retrieved. The data originate from our electronic medication administration system. In total, there were 48 variables.

#### Demographics

In total, there were two variables: age and sex.

#### Body composition (maximum 2 months before treatment)

In addition to weight, height and BMI, we included abdominal body composition, which was automatically obtained from CT images, to accurately assess the physical condition of patients. We retrieved abdominal CT images with a maximum interval of 2 months before treatment initiation and used a deep-learning model to automatically measure muscle, bone and different fat volumes (subcutaneous, visceral, intermuscular and total adipose tissue)^23^. The collected markers were divided by the number of abdominal CT slices to ensure patient comparability. In total, there were nine variables.

#### Cancer entity (C0-75)

For each patient, exactly one cancer entity was queried for which they were receiving treatment. In total, there were 60 variables.

#### Prior diagnoses (any before treatment)

We selected all ICD-10 codes (except C0-C75) that were present in at least 200 patients. In total, there were 68 variables.

#### Prior medical interventions (any before treatment)

We used the German operation and procedure classification system (OPS) to identify prior medical interventions. We selected all OPS codes that were present in at least 200 patients. In total, there were 50 variables.

#### Staging (maximum 1 year before treatment)

T, N and M status were obtained from tumor board documentation. In total, there were three variables.

#### Metastasis location (any before treatment)

Tissue affected by metastasis, if any, were included. In total, there were nine variables.

#### Vital signs (maximum 2 weeks before treatment)

Oxygen saturation, body temperature, heart rate and systolic and diastolic blood pressure were included. In total, there were five variables.

#### ECOG PS (maximum 3 months before treatment)

ECOG PS was obtained from tumor board documentation. In total, there was one variable.

#### Laboratory results (maximum 2 weeks before treatment)

We selected all variables that were present in at least 20% of patients (62 variables), plus nine others (mainly tumor markers) that we considered particularly relevant for subgroups. In total, there were 71 variables.

#### Pathology

Cancer subtype beyond ICD-10 classification, histologic tumor grade, immunohistochemical results and somatic tumor mutations were included. In total, there were 22 variables.

#### Smoking status

Smoking status (smoker/nonsmoker) and, if available, pack-years of smoking, were included. In total, there were two variables.

The endpoints OS and TTNT were automatically extracted from SHIP.

### Data preprocessing

Outliers, defined as >3 standard deviations from the mean, were removed for continuous variables. Continuous variables were prestandardized to zero mean and unit variance. Categorical scores were encoded on an ordinal scale (for example, ECOG PS as 0–4, metastasis as 0–1). Diagnoses (ICD codes), cancer entities, interventions (OPS codes) and systemic cancer treatments were one-hot encoded (0 = not present, 1 = present), which resulted in a total of 350 variables for the final dataset. For further analysis and description of differences between cancers, the cancer representations were summarized into more general cancer entities (Supplementary Table 1). To account for missing values while simultaneously keeping the ability to explain the present clinical markers, we applied feature expansion: $x\to (x,1-x)$. Missing values were set to $(\mathrm{0,0})$^50^. This method has been used previously in comparable biomedical settings^51,52^. Feature expansion was only applied to variables that had missing values. There were no missing values for ICD and OPS codes, systemic treatments, cancer diagnoses, age and sex.

### External Flatiron Health dataset

This study used the nationwide Flatiron Health electronic health record-derived deidentified database. The Flatiron Health database is a longitudinal database, comprising deidentified patient-level structured and unstructured data, curated via technology-enabled abstraction^53,54^. During the study period, the deidentified data originated from approximately 280 cancer clinics (~800 sites of care). The study included 3,288 patients diagnosed with advanced NSCLC from 1 January 2011 to 10 November 2022. The majority of patients (82.7%) originate from community oncology settings. The data are deidentified and subject to obligations to prevent reidentification and protect patient confidentiality. Patients with a birth year of 1937 or earlier may have an adjusted birth year in Flatiron datasets due to patient deidentification requirements.

For subsequent analysis in this study, extreme outliers were discarded manually before outliers, defined as >3 standard deviations from the mean, were removed for continuous variables. Further preprocessing of the data was performed analogously to the internal dataset, which resulted in a total of 18 variables for the final validation dataset.

### Model architecture

To model treatment outcomes, we used the coxph architecture similar to DeepSurv and the training regime from the pycox survival library^5,55^.

Each variable (potentially feature-expanded) was used as an input to a fully connected neural network with one hidden layer and a hidden width of 10 times the input neurons.

Thus, we decided to follow an early-fusion approach, as (1) all markers are one-dimensional and reasonably independent from each other (unlike, for example, pixels of an image or DNA sequences used in other studies) and (2) early fusion is particularly suitable for allowing interactions between markers^56^.

### Model training

Using five-fold cross-validation, we trained, for each fold, two neural networks (OS, TTNT) on 80% of the data to predict the proportional hazard risk score for OS and TTNT, respectively. We used the training algorithm supplied by the pycox library^55^. The remaining 20% of data was split randomly into a validation set (10%) to fine-tune the number of epochs and to early-stop the model and a test set (10%) for the computation of the concordance index. Cancer entities were balanced between training and validation/test sets for each fold. Model calibration was assessed using the python package *lifelines*^57^.

Models were trained for up to 50 epochs with a learning rate of 0.01 using the Adam optimizer. We used the default early stopping algorithm supplied by pycox. After the training process was early stopped, the learning rate was reduced to 1/10 of the previous learning rate and the model was trained for another 50 epochs. This was repeated down to a learning rate of 1e-4. We used a dropout rate of 0.5 and a batch size of 1024. To reduce the effect of correlations between input variables on the relevance explanation, we applied input dropout at a rate of 0.5 during training^48^. The concordance scores between predicted risk and ground truth were calculated for each fold using the pycox library. The identical training, validation, and test splits were used when neural networks were trained on individual cancer entities compared to training on the pan-cancer dataset to ensure comparability. Concordance results were discarded if the test set consisted of less than ten samples or if the test samples did not have at least five events.

### Explaining ML predictions

To explain the model’s predictions, we used LRP, a method for xAI that leverages the neural network structure of the model to compute explanations robustly and efficiently^12^. LRP starts with the prediction (the value obtained at the output of the neural network) redistributes it backwards, layer after layer, by means of propagation rules, and collects the explanation in the input layer. A physical analogy to the LRP propagation is water flowing through a network of pipes. In this physical network, the amount of water injected at the output equals the amount observed at the input.

More formally, let j and k be indices for neurons in two consecutive layers and ${a}_{j}$ and ${a}_{k}$ be their respective activations. In a typical neural network, including the DeepSurv network considered in this work, two consecutive layers are related generically by the equation:

$${a}_{k}=\rho\left(\mathop{\sum }\limits_{0,\,j}{a}_{j}{w}_{jk}\right)$$

In this equation, the sum runs over all neurons in the given layer plus a neuron with constant activation ${a}_{0}=1$. The variable ${w}_{{jk}}$ is the weight connecting neuron $j$ to neuron $k$. We then backpropagate using the generalized LRP-gamma rule, similar to previous works^51,52^. This rule propagates from one layer to the layer below using the equation:

$$\begin{array}{c}{R}_{j}={\sum }_{k}\frac{{a}_{j}^{+}\cdot \left({w}_{jk}+\gamma {w}_{jk}^{+}\right)+{a}_{j}^{-}\cdot \left({w}_{jk}+\gamma {w}_{jk}^{-}\right)}{{\sum }_{0,\,j}{a}_{j}^{+}\cdot \left({w}_{jk}+\gamma {w}_{jk}^{+}\right)+{a}_{j}^{-}\cdot \left({w}_{jk}+\gamma {w}_{jk}^{-}\right)}\cdot {1}_{{a}_{k} > 0}\cdot {R}_{k}\\\quad+{\sum }_{k}\frac{{a}_{j}^{+}\cdot \left({w}_{jk}+\gamma {w}_{jk}^{-}\right)+{a}_{j}^{-}\cdot \left({w}_{jk}+\gamma {w}_{jk}^{+}\right)}{{\sum }_{0,\,j}{a}_{j}^{+}\cdot \left({w}_{jk}+\gamma {w}_{jk}^{-}\right)+{a}_{j}^{-}\cdot \left({w}_{jk}+\gamma {w}_{jk}^{+}\right)}\cdot {1}_{{a}_{k} < 0}\cdot {R}_{k}\end{array}$$

where ${\left(.\right)}^{+}=\max (0,.)$ and ${\left(.\right)}^{-}=\min (0,.)$, and where $\gamma$ is a parameter that needs to be selected. Here, we used the heuristic 0.01, which worked well in other applications^52^. Applying the rule at each layer, starting at the top layer and moving backward until the input layer, we obtain in the last step the contribution of each input feature (that is, variable) to the prediction. For expanded features, the final LRP score is calculated as the sum of the LRP scores assigned to the tuple (x, 1 − x).

We treated the LRP score assigned to a specific input as the RC of this marker to the overall patient prognosis (OS or TTNT). The ‘marker importance’ of a marker across all patients was defined as the sum of the absolute LRP scores divided by the number of patients for whom this marker was not missing. To calculate the marker importance in a subcohort (for example, patients of a single cancer entity), LRP scores were first centered by subtracting the cohort mean.

### Statistics

No statistical methods were used to pre-determine sample sizes but our sample sizes are similar to those reported in previous publications. Data collection and analysis were conducted without randomization, and the investigators were not blinded to the conditions of the experiments. The statistical analyses were conducted in R statistical packages^58^. All tests were two-sided and results were regarded as significant if *P* < 0.05. Wilcoxon ranked test and Pearson correlation were computed using the package Hmisc^59^. Data distribution was assumed to be normal but this was not formally tested. A comparison of the xAI model to simplified models was done by first selecting the most important variables per fold (/and cancer type) using the CoxnetSurvivalAnalysis function (alpha = 0.9) from the python package *sksurv*^60^. Lambda was tuned to select 10 variables. Subsequently a linear Cox model was fitted on the reduced dataset. Linear regression was applied to fit relationships between marker values and their corresponding xAI-assigned RC for the internal and external datasets, respectively. Subsequently, the slope coefficients of these models were compared between the internal and external datasets.

The search for interactions between markers was quantified by comparing linear mixed-effects models with baseline models. For each marker pair, the relationship between the ‘primary’ marker and the RC was examined under the two conditions when the ‘secondary’ marker was high (highest 10%) or low (lowest 10%). For categorical variables, category levels were selected so that at least 10% of the samples were members of the high or low class, respectively. Medications, ICD codes, OPS codes, and cancer types were excluded from this analysis due to unbalanced levels. Marker pairs that were present in less than 100 samples were discarded. Holm’s multiple test correction was applied.

To validate marker relationships of higher complexity, we examined marker pairs that were found in the internal and external datasets. The difference in model coefficients between ‘high’ and ‘low’ classes was compared between both datasets. This analysis was restricted to markers that were present in both datasets. For the simple linear model, the baseline was a model consisting of the intercept only. For the mixed-effects linear model, the baseline consisted of a linear model with a fixed slope and a random intercept.

Additionally, these relationships between marker values and RC were compared with the coefficients (that is, hazard ratios) of univariate Cox proportional hazard models that predicted survival based on the respective markers. A mixed-effects variant of Cox proportional hazards models was used to validate the mixed-effects case. Cox models were discarded if they had a lower log-likelihood than their baseline models but did not have to be significant to be included in the comparison.

Cox proportional hazards models were implemented with the R package *survival*^61^. The mixed-effects variants of this analysis were modeled using the *coxme* package^62^. Other mixed-effects models were implemented with *lme4*^63^.

### Visualizations

Kaplan-Meier plots were computed with the R package *survival*^61^. Fig. 1 was created with BioRender.com (Klauschen^15^ BioRender.com/j46z292). All other plots were created with *ggplot2*^64^.

### Reporting summary

Further information on research design is available in the Nature Portfolio Reporting Summary linked to this article.

## Supplementary information

Supplementary Information Supplementary Table 1.

Reporting Summary

## Source data

Source Data Fig. 2 Statistical Source Data.

Source Data Fig. 3 Statistical Source Data.

Source Data Fig. 4 Statistical Source Data.

Source Data Fig. 5 Statistical Source Data.

Source Data Fig. 6 Statistical Source Data.

Source Data Fig. 7 Statistical Source Data.

Source Data Extended Data Fig. 2 Statistical Source Data.

Source Data Extended Data Fig. 3 Statistical Source Data.

Source Data Extended Data Fig. 4 Statistical Source Data.

Source Data Extended Data Fig. 5 Statistical Source Data.

Source Data Extended Data Fig. 6 Statistical Source Data.

Source Data Extended Data Fig. 7 Statistical Source Data.

Source Data Extended Data Fig. 8 Statistical Source Data.

Source Data Extended Data Fig. 9 Statistical Source Data.

Source Data Extended Data Fig. 10 Statistical Source Data.

## Data Availability

Data supporting the findings of the study are not publicly available due to privacy concerns, ethical considerations and legal requirements. Data cannot be shared with investigators outside the institution without consent. Access to anonymized data from University Hospital Essen may be granted for non-commercial research purposes, subject to a formal data access request and a case-by-case review process. Requests must include a detailed research plan and should be addressed to J. Kleesiek (Jens.Kleesiek@uk-essen.de) and will be forwarded to the relevant institutional review board within one month. Approved access requires the signing of a data use agreement. The external data have been originated by Flatiron Health, Inc. Requests for data sharing by license or by permission for the specific purpose of replicating results in this manuscript can be submitted to PublicationsDataAccess@flatiron.com. Access to Flatiron Health databases is subject to the execution of a data use agreement, which may include a use fee. Source data are provided with this paper.

## Code availability

Code is available at https://github.com/PhGK/DecodingCancer.
