# Supplementary material for: Decoding pan-cancer treatment outcomes using multimodal real-world data and explainable artificial intelligence
Source: Nat Cancer. 2025 Jan 30;6(2):307–22. doi: 10.1038/s43018-024-00891-1 (PMC11864985; doi:10.1038/s43018-024-00891-1)
Supplement: Supplementary file 1 — Supplementary Table 1. [file 43018_2024_891_MOESM1_ESM.pdf]

nature cancer

Supplementary information https://doi.org/10.1038/s43018-024-00891-1

Decoding pan-cancer treatment outcomes using multimodal real-world data and explainable artificial intelligence

In the format provided by the authors and unedited

Supplementary Table 1: Composition of the patient cohort.

Cancer	ICD	Patients
Lung	C34	4320
Sarcoma	C40, C41, C47-C49	1578
Breast	C50	1223
Head and Neck	C01-C13, C30-C32	1026
Liver	C22	728
Brain	C71	644
Pancreas	C25	620
Colon	C18	606
Melanoma	C43	600
Stomach	C16	545
Esophagus	C15	408
Eye	C69	384
Rectum	C20	373
Kidney	C64, C65	308
Uterus	C53-C55	275
Testis	C62	249
Prostate	C61	236
Mesothelioma	C45	229
Skin	C44	217
Bladder	C67	166
Biliary tract	C24	163
Ovary	C56	152
Thyroid gland	C73	117
Small intestine	C17	90
Heart	C38	84
Rectosigmoid junction	C19	80
Gallbladder	C23	64
Anus	C21	48
Other endocrine gland	C75	34

Urethra	C68	33
Vulva	C51	29
Other digestive organs	C26	24
Thymus	C37	21
Adrenal gland	C74	17
Penis	C60	11
Ureter	C66	10
Female genital	C57	9
Central nervous system	C72	5
