# Supplementary material for: Decoding pan-cancer treatment outcomes using multimodal real-world data and explainable artificial intelligence
Source: Nat Cancer. 2025 Jan 30;6(2):307–22. doi: 10.1038/s43018-024-00891-1 (PMC11864985; doi:10.1038/s43018-024-00891-1)
Supplement: Supplementary file 2 — Reporting Summary [file 43018_2024_891_MOESM2_ESM.pdf]

nature portfolio

Corresponding author(s): Jens Kleesiek

Last updated by author(s): Nov 21, 2024

Reporting Summary

Nature Portfolio wishes to improve the reproducibility of the work that we publish. This form provides structure for consistency and transparency in reporting. For further information on Nature Portfolio policies, see our Editorial Policies and the Editorial Policy Checklist.

Statistics

For all statistical analyses, confirm that the following items are present in the figure legend, table legend, main text, or Methods section.

n/a	Confirmed	
☐	☒	The exact sample size (*n*) for each experimental group/condition, given as a discrete number and unit of measurement
☐	☒	A statement on whether measurements were taken from distinct samples or whether the same sample was measured repeatedly
☐	☒	The statistical test(s) used AND whether they are one- or two-sided<br>*Only common tests should be described solely by name; describe more complex techniques in the Methods section.*
☐	☒	A description of all covariates tested
☐	☒	A description of any assumptions or corrections, such as tests of normality and adjustment for multiple comparisons
☐	☒	A full description of the statistical parameters including central tendency (e.g. means) or other basic estimates (e.g. regression coefficient) AND variation (e.g. standard deviation) or associated estimates of uncertainty (e.g. confidence intervals)
☐	☒	For null hypothesis testing, the test statistic (e.g. *F*, *t*, *r*) with confidence intervals, effect sizes, degrees of freedom and *P* value noted<br>*Give P values as exact values whenever suitable.*
☒	☐	For Bayesian analysis, information on the choice of priors and Markov chain Monte Carlo settings
☒	☐	For hierarchical and complex designs, identification of the appropriate level for tests and full reporting of outcomes
☐	☒	Estimates of effect sizes (e.g. Cohen's *d*, Pearson's *r*), indicating how they were calculated

Our web collection on statistics for biologists contains articles on many of the points above.

Software and code

Policy information about availability of computer code

Data collection	Data was collected from our database using Python 3.8.8
Data analysis	Neural network models were fitted using torch 2.1.1, pycox 0.2.3 and torchtuples 0.2.2. Survival analyses were conducted using scikit-learn 1.3.2 and scikit-survival 0.22.2 in python 3.11.6, and survival 3.5.8 and survcomp 1.38.0 in R 4.0.3. Statistical tests were performed using Hmisc 4.7.2 and stats 4.0.3. Linear models were built using glmnet 4.1.8, lme4 1.1.35.1, forecast 8.21.1, and coxme 2.2.18.1.<br>Code is available at: https://github.com/PhGK/DecodingCancer

For manuscripts utilizing custom algorithms or software that are central to the research but not yet described in published literature, software must be made available to editors and reviewers. We strongly encourage code deposition in a community repository (e.g. GitHub). See the Nature Portfolio guidelines for submitting code & software for further information.

Data

Policy information about availability of data

All manuscripts must include a data availability statement. This statement should provide the following information, where applicable:

- Accession codes, unique identifiers, or web links for publicly available datasets
- A description of any restrictions on data availability
- For clinical datasets or third party data, please ensure that the statement adheres to our policy

Data supporting the findings of the study are not publicly available due to privacy concerns, ethical considerations and legal requirements. Data cannot be shared

with investigators outside the institution without consent. Access to anonymized data from University Hospital Essen may be granted for non-commercial research purposes, subject to a formal data access request and a case-by-case review process. Requests must include a detailed research plan and should be addressed to J. Kleesiek (Jens.Kleesiek@uk-essen.de) and will be forwarded to the relevant institutional review board within one month. Approved access requires the signing of a data use agreement.
The external data have been originated by Flatiron Health, Inc. Requests for data sharing by license or by permission for the specific purpose of replicating results in this manuscript can be submitted to PublicationsDataAccess@flatiron.com. Access to Flatiron Health databases is subject to the execution of a data use agreement, which may include a use fee. Source data are available with this paper.

Research involving human participants, their data, or biological material

Policy information about studies with human participants or human data. See also policy information about sex, gender (identity/presentation), and sexual orientation and race, ethnicity and racism.

Reporting on sex and gender	Sex and gender did not have any impact on patient selection.
Reporting on race, ethnicity, or other socially relevant groupings	Race or ethnicity did not have any impact on patient selection.
Population characteristics	All patients with solid tumors were collected based on ICD codes. Then, patients who received intravenous or oral cancer treatment documented in our Hospital Information Platform were selected. Further inclusion criteria were: Initiation of systemic therapy since April 2007 and a minimum age of 18 years at the initiation of cancer treatment. A detailed overview of the patient enrollment process can be found in the supplementary material.
Recruitment	We retrospectively evaluated data from 150,079 cancer patients with available medical records treated at University Hospital Essen. Of these, 15,726 patients who received systemic cancer treatment between April 2007 and July 2022 were included in the final analysis.
Ethics oversight	The study was approved by the Ethics Committee of the Medical Faculty of the University of Duisburg-Essen (No. 21-10347-BO). The requirement for written informed consent was waived due to the retrospective design of the study and the de-identification of data.

Note that full information on the approval of the study protocol must also be provided in the manuscript.

Field-specific reporting

Please select the one below that is the best fit for your research. If you are not sure, read the appropriate sections before making your selection.

☒ Life sciences ☐ Behavioural & social sciences ☐ Ecological, evolutionary & environmental sciences

For a reference copy of the document with all sections, see nature.com/documents/nr-reporting-summary-flat.pdf

Life sciences study design

All studies must disclose on these points even when the disclosure is negative.

Sample size	Electronic health records from 150,079 cancer patients treated at the University Hospital Essen were retrospectively evaluated. Of these, 15,726 patients who received systemic cancer treatment for a solid malignancy at University Hospital Essen between April 2007 and July 2022 and had a minimum age of 18 years were included in this study. No statistical methods were used to pre-determine sample sizes but our sample sizes are similar to those reported in previous publications.
Data exclusions	A detailed overview of the patient enrollment process can be found in the Extended Data Figure 1.
Replication	We performed multiple independent experiments. Results were consistent and all experiments were reproducible.
Randomization	Due to the retrospective study design, no randomization was performed.
Blinding	Blinding was not applicable as our study is a retrospective data analysis from electronic health records.

Reporting for specific materials, systems and methods

We require information from authors about some types of materials, experimental systems and methods used in many studies. Here, indicate whether each material, system or method listed is relevant to your study. If you are not sure if a list item applies to your research, read the appropriate section before selecting a response.

Materials & experimental systems

n/a	Involved in the study
☒	☐ Antibodies
☒	☐ Eukaryotic cell lines
☒	☐ Palaeontology and archaeology
☒	☐ Animals and other organisms
☒	☐ Clinical data
☒	☐ Dual use research of concern
☒	☐ Plants

Methods

n/a	Involved in the study
☒	☐ ChIP-seq
☒	☐ Flow cytometry
☒	☐ MRI-based neuroimaging

Plants

Seed stocks	Not applicable
Novel plant genotypes	Not applicable
Authentication	Not applicable
